# Supplementary material for: High matrix metalloproteinase-2 expression predicts poor prognosis of colon adenocarcinoma and is associated with PD-L1 expression and lymphocyte infiltration
Source: PeerJ. 2025 Jun 30;13:e19550. doi: 10.7717/peerj.19550 (PMC12225630; doi:10.7717/peerj.19550)
Supplement: Supplemental Information 2 [file peerj-13-19550-s002.pdf]

Original blots and images:

Original blots Western blots of knockdown-MMP-2 and PD-L1 under the intervention of MMP-2 in SW480 cells.

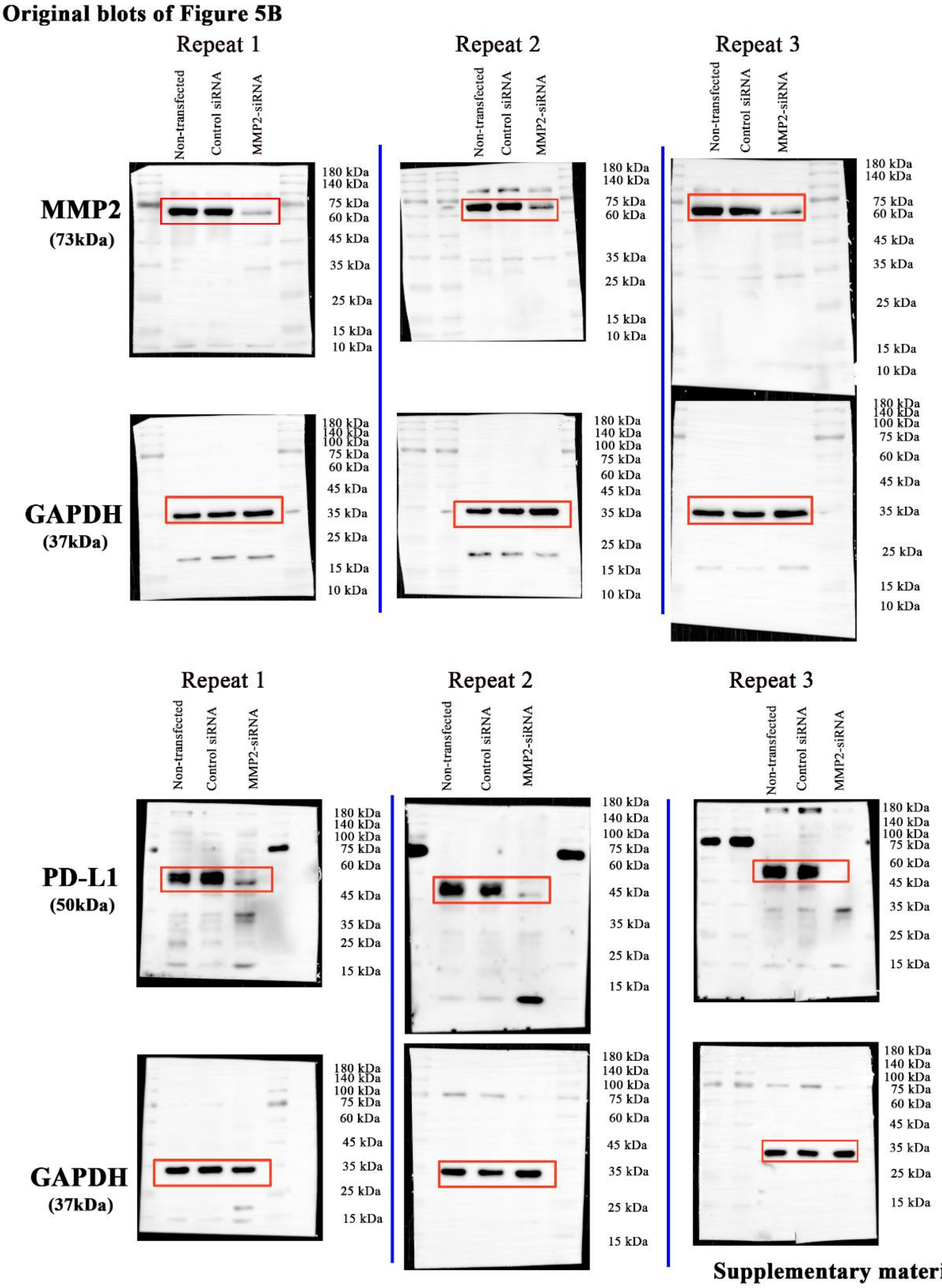

Supplementary material
