# Supplementary figures and images for: High matrix metalloproteinase-2 expression predicts poor prognosis of colon adenocarcinoma and is associated with PD-L1 expression and lymphocyte infiltration
Source: PeerJ. 2025 Jun 30;13:e19550. doi: 10.7717/peerj.19550 (PMC12225630; doi:10.7717/peerj.19550)

Original blots of Supplementary Figure 4

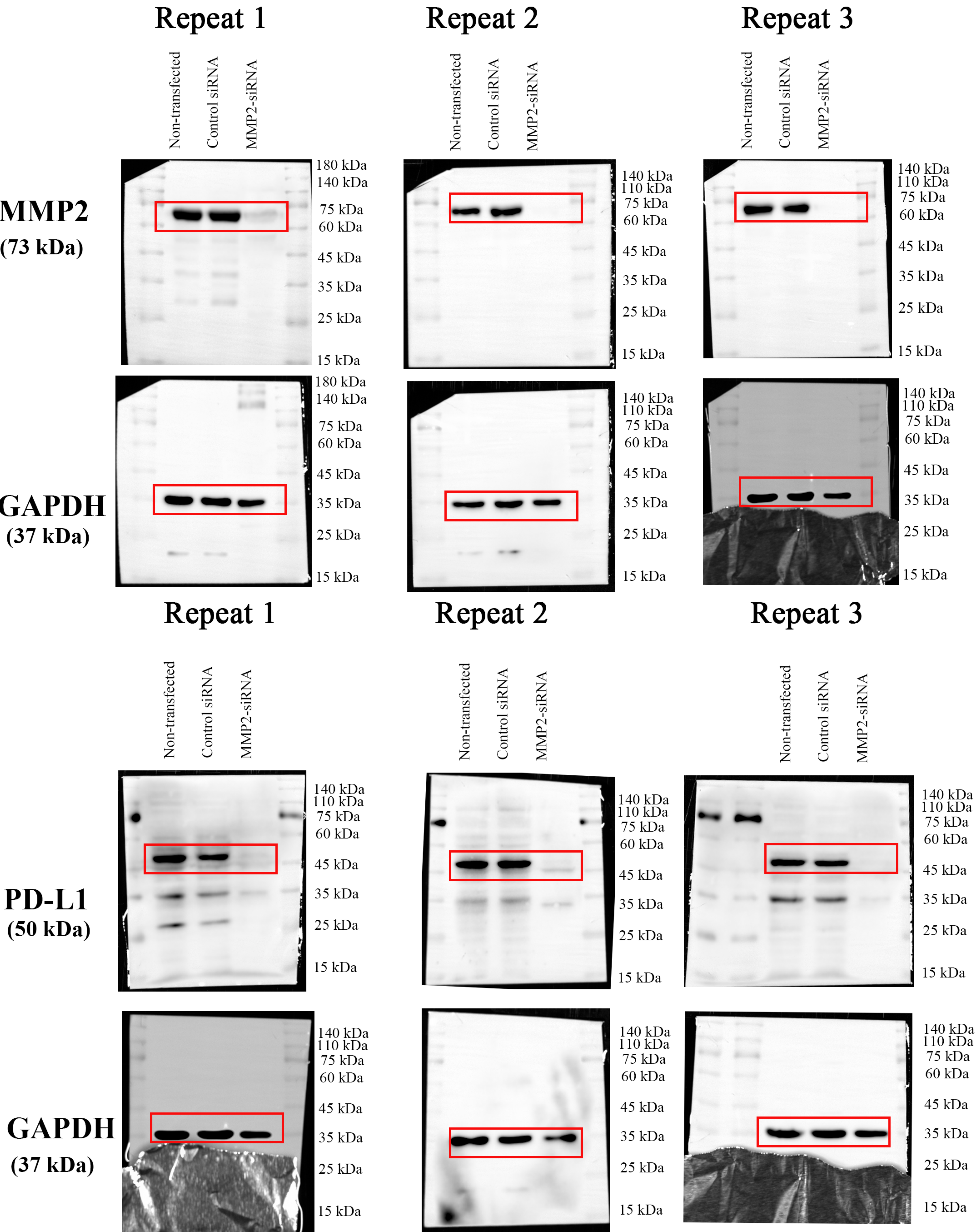

# Original blots of Supplementary Figure 6

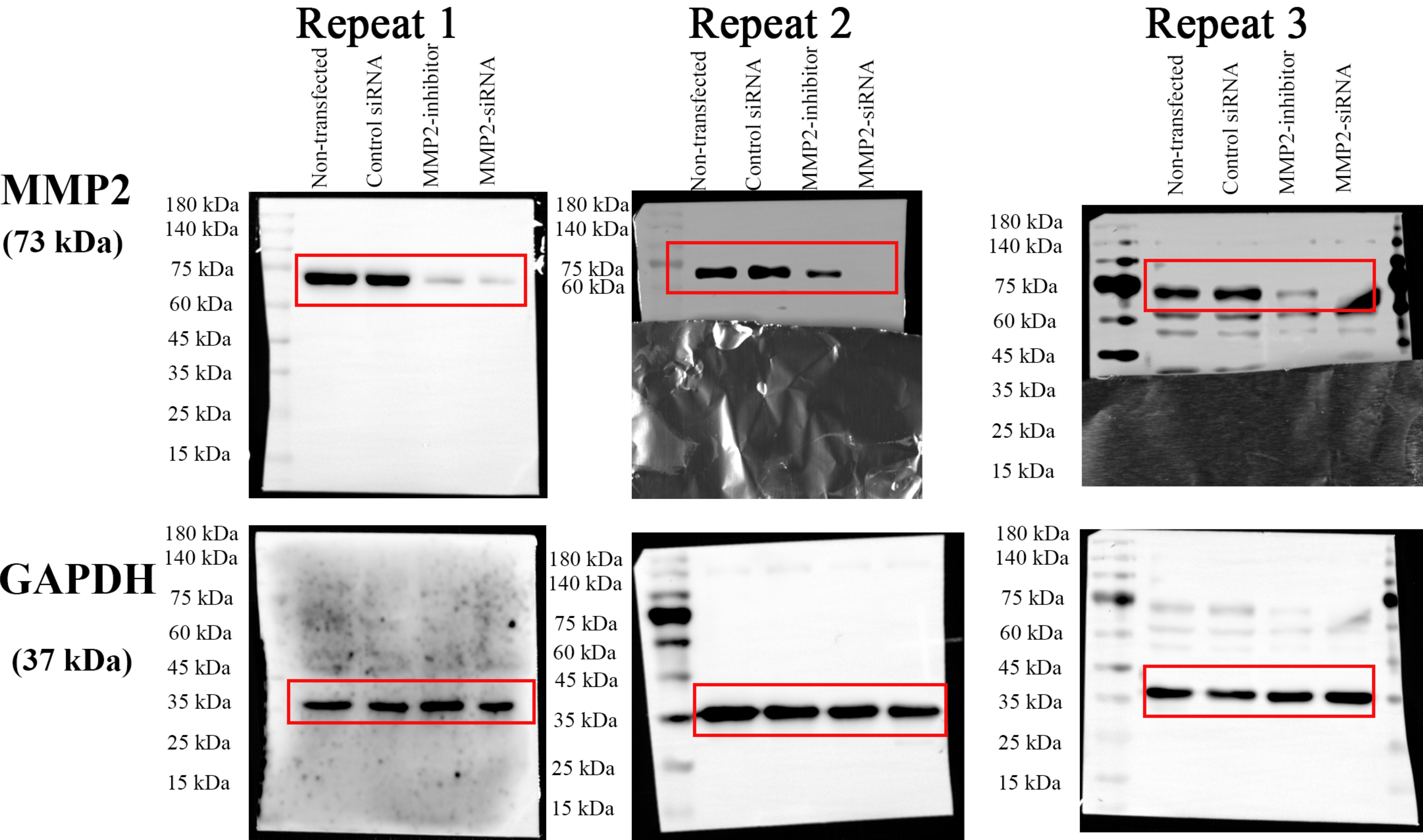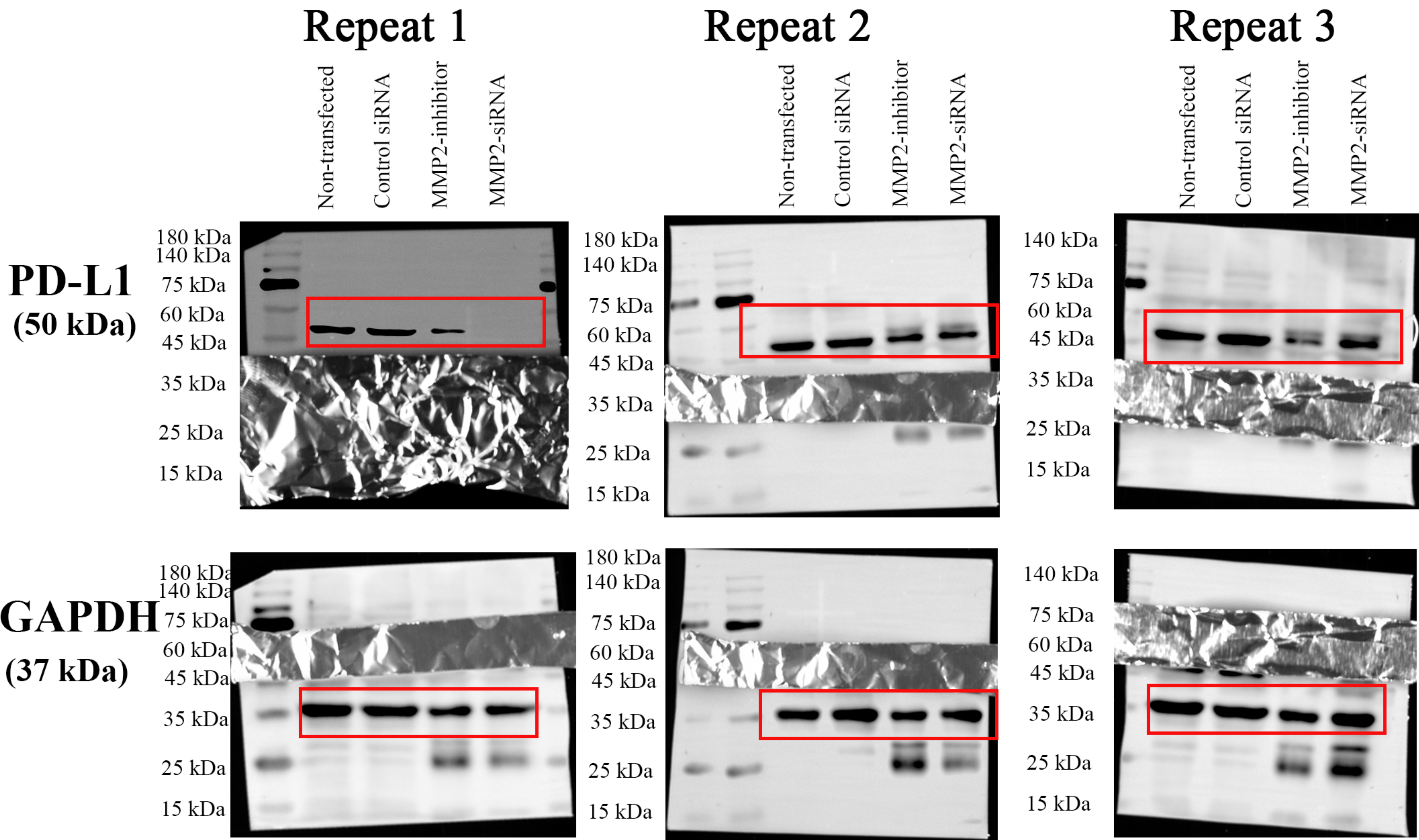

Supplement: Supplemental Information 8 — Uncropped blots of MMP2, PD-L1, and GAPDH protein. [file peerj-13-19550-s008.pdf]

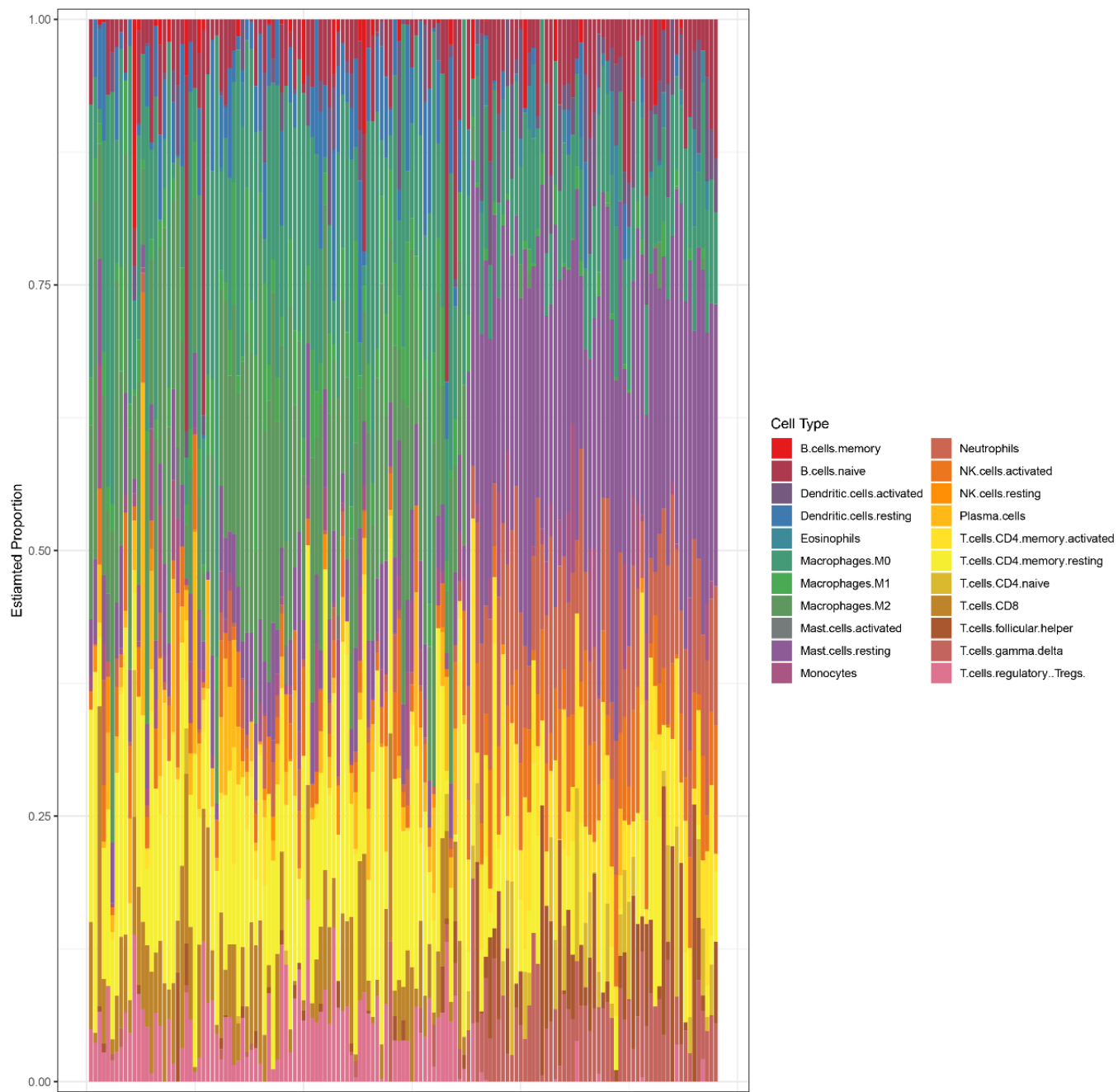

Supplement: Supplemental Information 9 — The horizontal axis represents different patients, and the vertical axis represents different proportions of infiltrating immune cells. [file peerj-13-19550-s009.pdf]

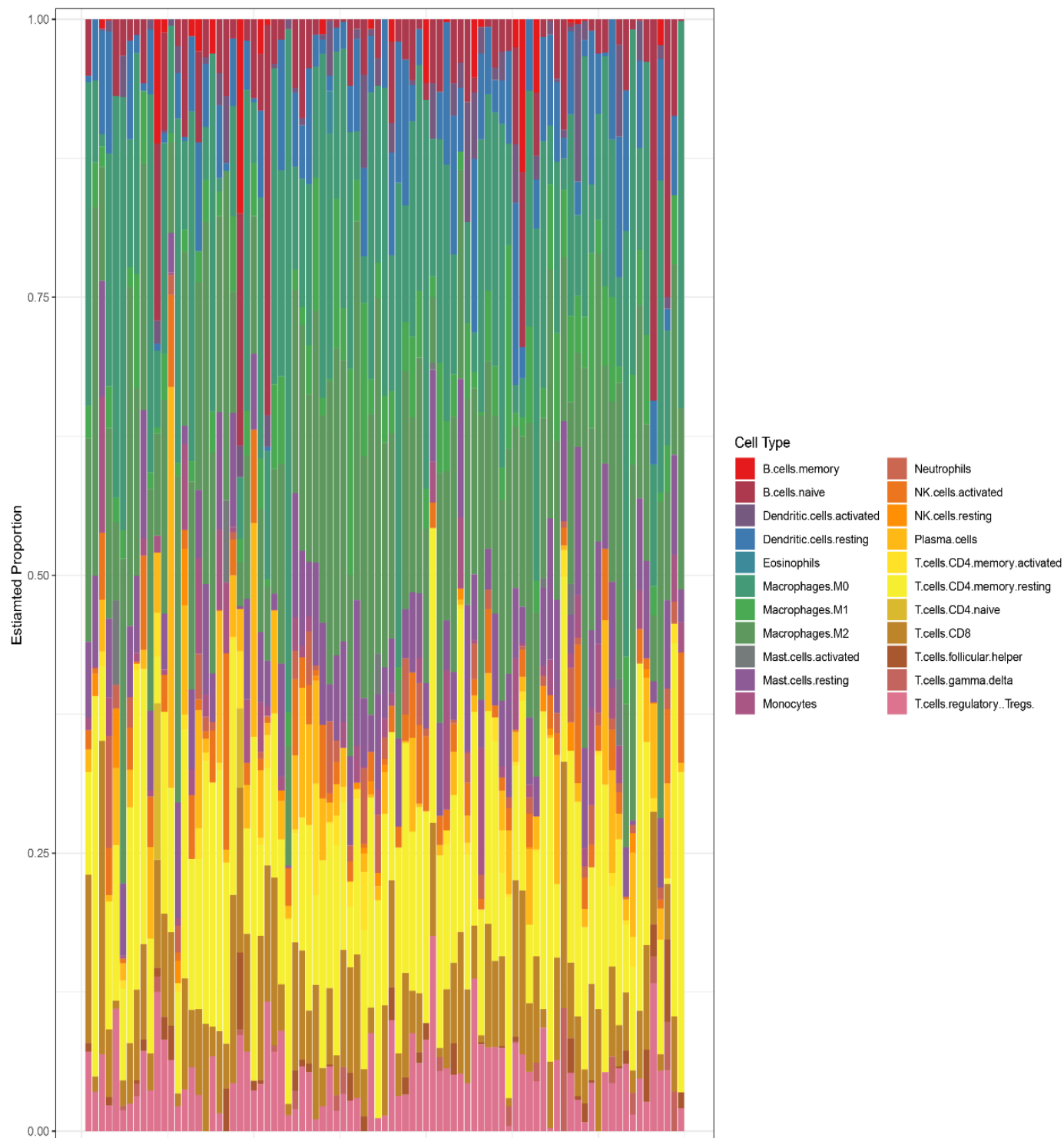

Supplement: Supplemental Information 10 — The horizontal axis represents different patients, and the vertical axis represents different proportions of infiltrating immune cells. [file peerj-13-19550-s010.pdf]

A

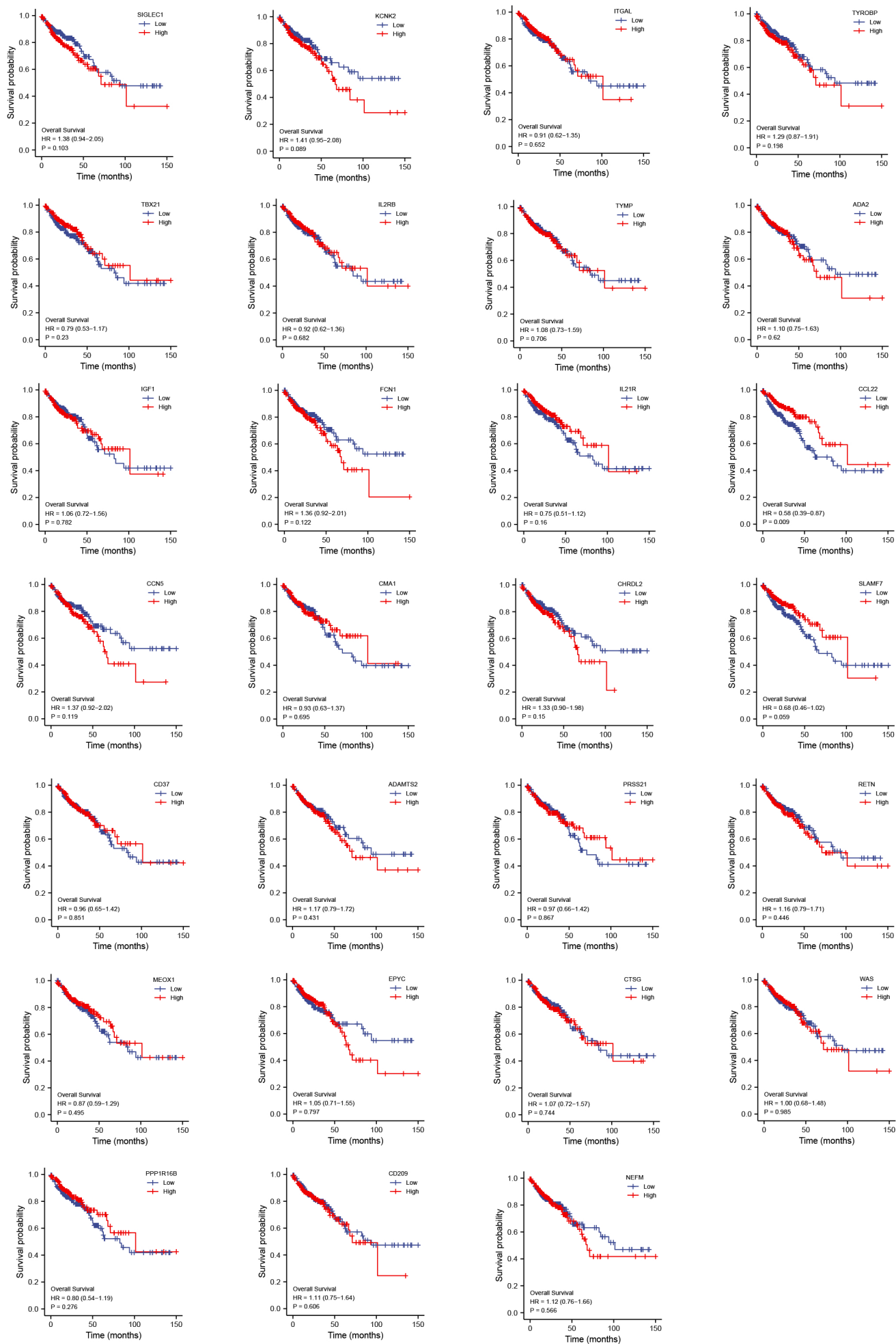

Supplement: Supplemental Information 11 — The remaining hub genes from Figure 3B are presented here. The red line indicates higher gene expression, and the blue line indicates lower gene expression. [file peerj-13-19550-s011.pdf]

A

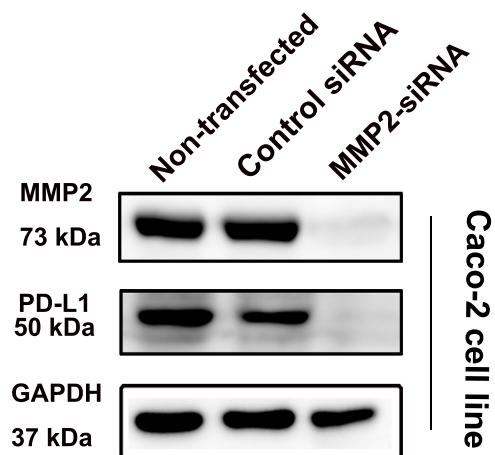

B

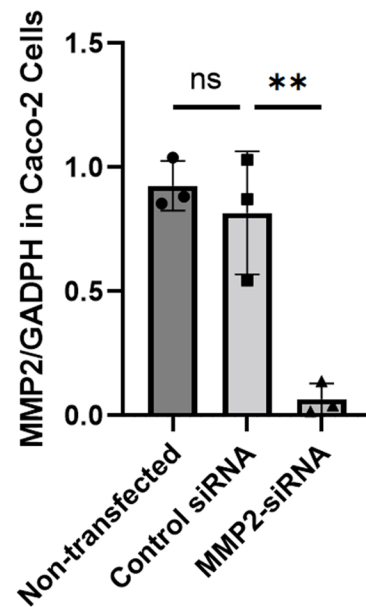

C

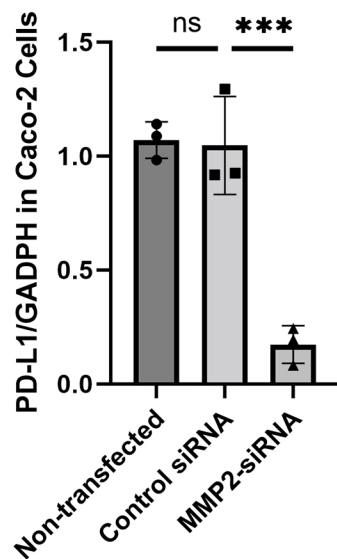

Supplement: Supplemental Information 12 — (A) Representative Western blot images showing MMP2 and PD-L1 protein levels in Caco-2 cells transfected with Control siRNA or MMP2-targeting siRNA (MMP2-siRNA). (B) Quantification of MMP2 expression relative to GAPDH in Control siRNA and MMP2-siRNA groups, n = 3/group. (C) Quantification of PD-L1 expression relative to GAPDH in Control siRNA and MMP2-siRNA groups, n = 3/group. **p < 0.01, ***p < 0.001, compared to the Control siRNA group. [file peerj-13-19550-s012.pdf]

A

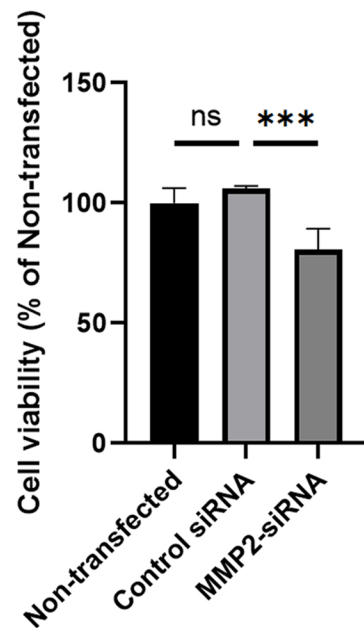

C

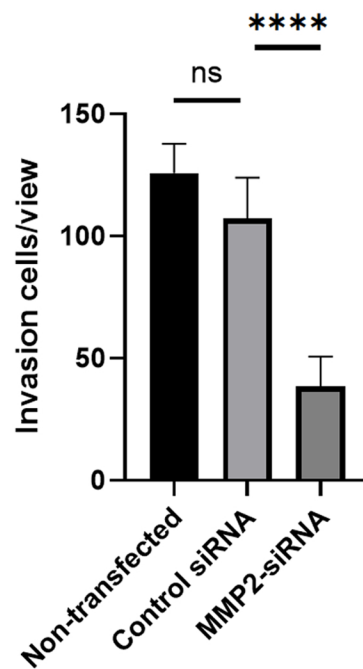

B

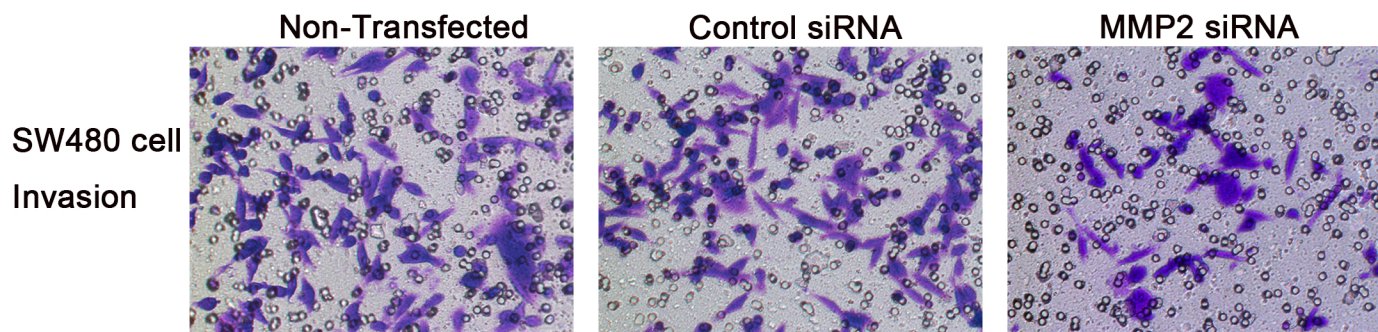

Supplement: Supplemental Information 13 — (A) CCK-8 assay showing cell proliferation in Non-transfected, Control siRNA, and MMP2-siRNA transfected SW480 cells. (B) Transwell invasion assay showing the invasive capacity of Non-transfected, Control siRNA, and MMP2-siRNA transfected SW480 cells, magnification of ×200. (C) Average invasive cell number per field. ***p<0.001, ****p<0.0001, ns, not significant (p>0.05), compared to Control siRNA. [file peerj-13-19550-s013.pdf]

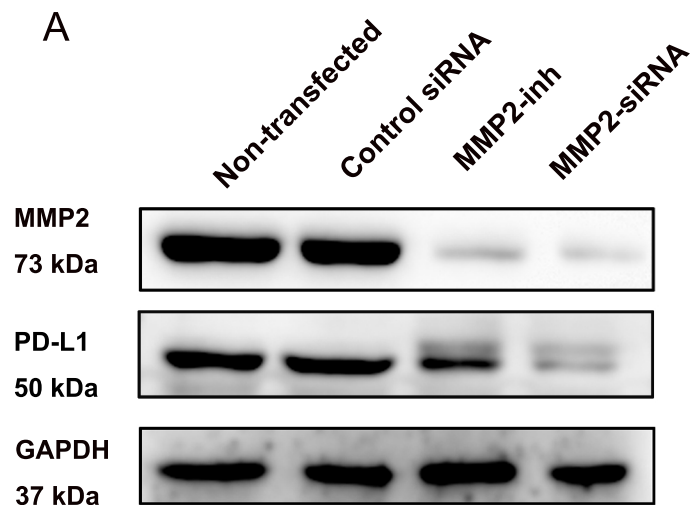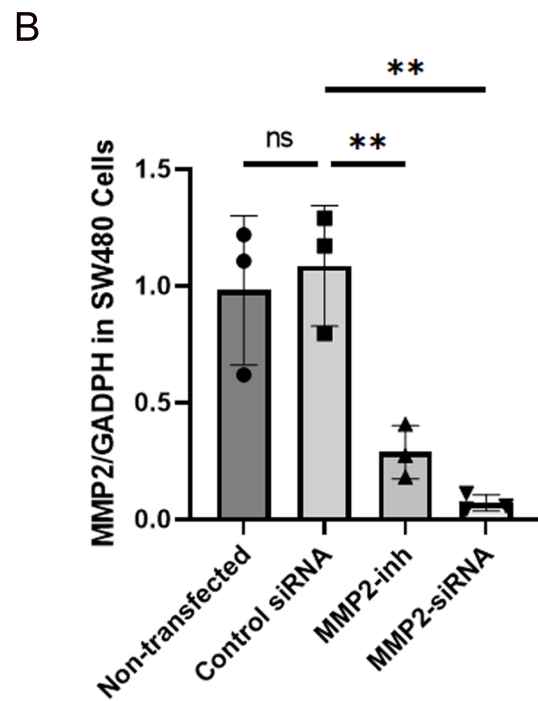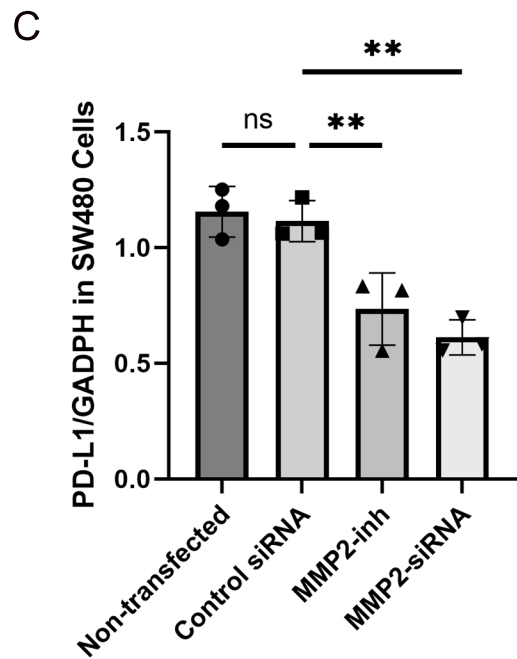

Supplement: Supplemental Information 14 — (A) SW480 cells were treated with MMP-2 selective inhibitor SB-3CT (25 μM, MMP2-inh group), the level of MMP-2 and PD-L1 were detected by Western blot. (B) Relative protein expression of MMP-2, n=3/group. (C) Relative protein expression of PD-L1, n=3/group. **p < 0.01, ns, not significant (p>0.05), compared to Control siRNA. [file peerj-13-19550-s014.pdf]
